# Differential Methylation of H3K79 Reveals DOT1L Target Genes and Function in the Cerebellum In Vivo

**DOI:** 10.1007/s12035-018-1377-1

**Published:** 2018-10-10

**Authors:** Patrick Piero Bovio, Henriette Franz, Stefanie Heidrich, Tudor Rauleac, Fabian Kilpert, Thomas Manke, Tanja Vogel

**Affiliations:** 1 Institute for Anatomy and Cell Biology, Department of Molecular Embryology, Medical Faculty, University of Freiburg, 79104 Freiburg, Germany; 2 Faculty of Biology, University of Freiburg, 79104 Freiburg, Germany; 3 Max Planck Institute of Immunobiology and Epigenetics, 79108 Freiburg, Germany

**Keywords:** Atoh1, Purkinje cell, Ataxia, Epigenetics

## Abstract

The disruptor of telomeric silencing 1-like (DOT1L) mediates methylation of histone H3 at position lysine 79 (H3K79). Conditional knockout of *Dot1l* in mouse cerebellar granule cells (*Dot1l*-cKO^*Atoh1*^) led to a smaller external granular layer with fewer precursors of granule neurons. *Dot1l*-cKO^*Atoh1*^ mice had impaired proliferation and differentiation of granular progenitors, which resulted in a smaller cerebellum. Mutant mice showed mild ataxia in motor behavior tests. In contrast, Purkinje cell-specific conditional knockout mice showed no obvious phenotype. Genome-wide transcription analysis of *Dot1l*-cKO^*Atoh1*^ cerebella using microarrays revealed changes in genes that function in cell cycle, cell migration, axon guidance, and metabolism. To identify direct DOT1L target genes, we used genome-wide profiling of H3K79me2 and transcriptional analysis. Analysis of differentially methylated regions (DR) and differentially expressed genes (DE) revealed in total 12 putative DOT1L target genes in *Dot1l*-cKO^*Atoh1*^ affecting signaling (*Tnfaip8l3, B3galt5*), transcription (*Otx1*), cell migration and axon guidance (*Sema4a*, *Sema5a*, *Robo1*), cholesterol and lipid metabolism (*Lss*, *Cyp51*), cell cycle (*Cdkn1a*), calcium-dependent cell-adhesion or exocytosis (*Pcdh17*, *Cadps2*), and unknown function (*Fam174b*). Dysregulated expression of these target genes might be implicated in the ataxia phenotype observed in *Dot1l*-cKO^*Atoh1*^.

**Electronic supplementary material:**

The online version of this article (10.1007/s12035-018-1377-1) contains supplementary material, which is available to authorized users.

## Introduction

The cerebellum coordinates mainly sensorimotor functions. Moreover, its proper function is affected in various neurological diseases, including ataxia [1], autism [2], schizophrenia [3], or Angelman syndrome [4]. But the cerebellar network has various additional roles, and recent observations report on cerebellar functions in cognition and emotion [5]. One possible cause for cerebellar dysfunction is disturbance of its development and alterations in proliferation, migration, neuronal differentiation, and/or synaptic function.

The cerebellum develops from two different germinal zones. The neuroepithelium lining the fourth ventricle gives rise to the Purkinje cells (PC). These neurons have inhibitory properties and are the main efferent projection neurons of the cerebellum. A second germinal zone is the external granular layer (EGL), in which the cerebellar granule cell precursors (CGNP) reside. The CGNP of the EGL gives rise to the cerebellar granule neurons (CGN), which migrate from the outer ridge inside, crossing the molecular and PC layer. Neuronal differentiation is occurring during migration, and mature neurons finally settle within the internal granular layer (GL) of the developing cerebellum [6]. CGN are one of the most abundant fractions of neurons within the central nervous system [7] and receive afferent inputs from mossy fibers of diverse anatomical locations.

Epigenetic modifications of DNA or histones control transcription in a variety of cellular contexts, also during formation of the brain. DNA methylation and histone acetylation are the most intensive studied epigenetic modifications in brain development [8, 9]. Recent studies report on the emerging role of histone methylations [10]. However, little is known about the implication of histone modifications specific for the development and function of the cerebellum. It has been reported that DNA methylation and hydroxymethylation [11] as well as chromatin remodeling [12] are important for granule cell function and development. But ChIP-seq data are lacking for histone methylation or acetylation as yet.

We recently reported on the role of MLLT3/AF9 and DOT1L in cell specification in the cerebral cortex [13, 14]. MLLT3/AF9 binds acetylated histones and interacts with DOT1L, which is mediating histone H3 mono-, di-, and trimethylation at position 79 (H3K79me1, H3K79me2, H3K79me3) [15]. H3K79 methylation is mainly considered transcriptional activating when highly abundant [16], although association with transcriptionally repressed loci has also been observed [13, 17]. Human patients presenting with complex neurological symptoms, including ataxia, revealed genomic deletions that included the MLLT3/AF9 locus [18]. We therefore hypothesized that DOT1L and H3K79 methylation might be of relevance for the development and/or function of the cerebellum.

We here report on histological and behavioral characterization of DOT1L-deficient mice. DOT1L deficiency in granule cells, but not in PCs, led to an ataxia phenotype and a smaller sized cerebellum in mice. We studied cell proliferation and differentiation in in vitro cultured CGNP and CGN under pharmacological inhibition of DOT1L activity. These analyses revealed that DOT1L activity is needed for proper cell proliferation of CGNP. Microarray analysis of transcriptional alterations and H3K79me2 ChIP-seq identified differentially expressed genes (DE) as well as differentially methylated regions (DR) in either CGNP or CGN after pharmacological interference with DOT1L activity. The data analysis revealed that impaired DOT1L activity affected a variety of important developmental and homeostatic processes in the cerebellum. Derailed expression of DOT1L target genes was confirmed in vivo in *Dot1l*-cKO mice. In summary, this study reports on DOT1L target genes in the cerebellum that might account for the ataxia phenotype in mice with DOT1L-deficient granular cells in the cerebellum.

## Material and Methods

### Mice

Experiments were performed following the European Communities Council Directive and were approved by animal welfare committees of the University of Freiburg as well as local authorities (X-17/03S and G12/03). CGN and CGNP cultures derived from NMRI P7 mice. E18.5, P0, P3, or week (W) 9 conditional KO mice with C57BL6/J background harbored floxed *Dot1l* at exon 2 leading to frame shift resulting in a non-functional gene product. The *Dot1l* mouse line was obtained from the Knockout Mouse Project (KOMP). cKO mice are as follows: *Dot1l*^flox/flox^, *Atoh1*^cre/+^ or *Dot1l*^flox/flox^, *Pcp2*^cre/+^. Control mice are as follows: *Dot1l*^flox/flox^ or ^flox/+^, *Atoh1*^+/+^ or *Dot1l*^flox/flox^ or ^flox/+^, *Pcp2*^+/+^. *Pcp2*^cre/+^ and *Atoh1*^cre/+^ mouse lines are described elsewhere [19, 20].

### Real-time Cell Analysis and Inhibitor Treatment for DOT1L

Real-time cell analysis (RTCA) was performed with xCELLigence (ACEA Bioscience, SD, USA) as described in [14]. 2 × 10^5^ cells were used per well. The “normalized cell index” compares the change of impedance due to cellular coverage on a 16-well E-plate (Roche, Swiss) to a medium-only control. For inhibitor treatment, 5 μM SGC0946 (Sigma-Aldrich, MO, USA) or EPZ5676 (Absource Diagnostics, Germany), solved in dimethyl sulfoxide (DMSO, Sigma-Aldrich, MO, USA), was applied to CGNP 20 h after plating, for a duration of 4 h or 24 h. To CGN, the inhibitor was applied 4 h after plating, for the duration of 44 h. During RTCA, SGC0946 was replenished every 48 h with half a media change.

### Primary CGNP and CGN Culture, Immunocytochemistry, and Cell Counting

CGNP and CGN cultures were kept as described [21]. Immunocytochemistry (ICC) was performed as described [22–24]. For ICC immunostainings, CGNP or CGN were plated with a density of 5 × 10^5^ cells/24-well. Antibodies used were aCASP3 (asp175, Cell Signaling, MA, USA), KI67 (ab15580, Abcam, Cambridge, UK), and HuC/D (16A11, Invitrogen, PA, USA). Per biological replicate and condition, 10 to 15 fields (× 200 magnification) were counted for the respective antibody and DAPI using the software FiJi (FiJi is ImageJ) [25], including the nucleus counter macro for particle analysis in CookBook (http://imagej.net/Cookbook) [26]. The experimenter was blind for the treatment condition during quantification of images.

### Quantitative Real-time PCR and Immunoblot

qRTPCR and immunoblots were performed as described [24]. Immunoblots were quantified with FiJi [25]. Statistics are as follows: qRTPCR—mean log_2_FC ± *SEM* with two-sided *t* test and equal variance, *p ≤ 0.05, **p ≤ 0.005, ***p ≤ 0.0005; WB—one-sample *t* test and equal variance, *p ≤ 0.05, **p ≤ 0.005, ***p ≤ 0.0005.

### Immunohistochemistry (IHC) and Quantification

For E18.5, P0, P3, and P7 animals, cerebelli were fixed overnight in 4% PFA directly after dissection. Adult animals were perfused with 4% PFA followed by overnight fixation of the tissue at 4 °C, PBS washes, overnight incubation in 30% sucrose/PBS at 4 °C, and embedding in Tissuetec (Leica, Richmond, IL, USA) before cryosectioning into 40-μm sagittal sections. Six serial sections per cerebellum were transferred into cryoprotection solution (0.1 M sodium phosphate buffer pH 7.2, *w*/*v* 30% sucrose, *v*/*v* 1% PVP-40, 30% ethylene glycol) and stored at − 20 °C. For IHC, sections were washed in PBS and thrice in TBS (pH 9) for 5, 10, and 15 min before antigen retrieval at 90 °C for 20 min. Washing was performed thrice in 0.1% Triton-X/PBS for 5, 10, and 15 min before blocking in 5% BSA/5% NDS/0.1% Triton-X/PBS for 4 h. Sections were incubated in blocking solution containing 0.1% sodium azide and primary antibody of desired dilution for 48 to 72 h at 4 °C, subsequently washed thrice in 0.1% Triton-X/PBS as above, incubated with secondary antibody (1:500 Alexa 488/568/594, donkey, Dianova, Hamburg, Germany) for 3 h, washed as above, and DAPI staining was performed for 10 min. Washed sections were mounted on gelatine/chromalaun-coated glass slides with fluorescent mounting medium (#S3023, DAKO, Jena, Germany). Antibodies used were NEUN (MAB377, Millipore, Darmstadt, Germany), CALB2 (ab92341, Abcam), KI67 (ab15580, Abcam), BrdU (ab1893, Abcam), PAX6 (PRB-278P, Covance, TX, USA), CALB1-D-28K (c9848, Sigma-Aldrich), and MEIS1 (ab19867, Abcam).

For counting cells per millimeter length of EGL, a ROI of 1000 ± 200 μm alongside the VIa lobe of at least three midsagittal sections per biological replicate with a minimum distance between sections of 240 μm was quantified and normalized to 1000 μm length of EGL [17]. For counting cells per 0.1 mm^2^ of GL, a ROI of 0.1 ± 0.05 mm^2^ was used in the area next to the quantified length of EGL [7]. The experimenter was double blind for the genotype during quantification of images.

### Behavioral Tests on Balance Beam

Experimenter was blinded to genotypes. On the first day, 9- to 10-week (W) old male mice were trained to traverse a squared beam of 2 cm diameter by placing the animal 90° to the beam in the middle of it (Fig. S2D). The animal had time to traverse the beam for max. 2 min. On the second day, the mice were placed on three different beams for 2 min each with increasing challenge from squared beams with decreasing diameters of 1.5 cm and 0.5 cm, to a round beam with a diameter of 0.5 cm. In between the challenges, the mice rested for 1 h and each session was video recorded. Segments crossed and number of hind leg slipping were quantified. Outlier were defined if they deviated ≥ 2.5 × from the ± *SEM* and removed from the dataset before statistical analysis. We defined the null hypothesis (H0) that mutant mice motor performance was equal to (=) ctrl mice motor performance and the hypothesis 1 (H1) that mutant mice motor performance is worse than (<) ctrl mice motor performance. Accordingly, a one-sided *t* test, unpaired, with equal variance was used for statistical analysis. Significant levels were indicated by *p ≤ 0.05, **p ≤ 0.005, and ***p ≤ 0.0005.

### ChIP

ChIP procedure was performed with two biological replicates as described [21]. Antibodies used for ChIP were H3K79me2 (ab3594, Abcam) and IgG (C15410206, Diagenode, Seraing, Belgium).

### Bioinformatic Analysis

Affymetrix microarrays (Mouse Gene ST 2.0 arrays) were used. Datasets are available at gene expression omnibus (GEO) under the following accession number: GSE101945. For microarray data analysis, Partek Genomics Suite software was used (Partek Inc., MO, USA). After interrogating probes, pre-background adjustment for GC content and probe sequence, and RMA background correction, the array data were normalized using quantile normalization and probe set summarization using Median Polish. Probe values were log_2_ transformed. The median or the Tukey biweight method was used to calculate the gene summary for each transcript cluster ID. Differentially expressed genes between the groups were determined by one-way ANOVA. Using Fisher’s least significant difference (LSD), contrasts between the groups were performed. Significance level (*p*-value) was corrected for false discovery rate (FDR) to a step-up *p*-value.

For ChIP-seq analysis, quality control was performed with FastQC, control for CG bias with computeCGBias, and for ChIP to input efficiency with bamCorrelate and bamFingerprint as part of deepTools2 [27, 28]. UCSC-main RefSeq GRCm38/mm10 was used as reference genome for Bowtie2 mapping and plotting [29]. Bowtie2 [27, 30] was used for mapping and duplicates were removed by MarkDuplicates (http://broadinstitute.github.io/picard/) by default settings. Further information about mapping quality calculated with flagStat and Bowtie2 as well as FastQC can be found as MultiQC in addition to information about size distribution in metricChIPseq on our GitHub repository (https://github.com/pbovio/CGNP-CGN-SES-H3K79me2-Bovio). Data was normalized using signal extraction scaling (SES) [31, 32] before calculating the log_2_(ratio of the number of reads) of ChIP to input by bamCompare using a bin size of 25 bp and a pseudocount of 1. For plotting, computeMatrix and plotHeatmap including k-mean clustering of deepTools2 tool shed was used. The ChIP-seq tools were used on the Galaxy Platform (https://galaxy.uni-freiburg.de/) [22]. Data is available at GEO: GSE101947.

For both biological replicates of ChIP datasets, DiffBind v2.2 [33] was used to define differentially methylated regions from MACS2 v2.1 [34, 35] broad peak calls. Threshold *q*-value of 0.1 was used for MACS2 broad peak and DiffBind. HOMER v4.9 [36] was used for GO term analysis. Enrichment was calculated assuming the cumulative hypergeometric distribution which is corresponding to Fisher’s exact test.

To compare DE genes and DR regions, IPython Jupyter notebook under Python3 with matplotlib, NumPy, SciPy, PyLab, and pandas packages as well as Galaxy was applied. Plotting of heatmaps and Venn diagram Python3 was used. Statistical power calculations and screening for minimal effect size were done with R. For detailed Galaxy history and IPython Jupyter notebook, see GitHub (https://github.com/pbovio/CGNP-CGN-SES-H3K79me2-Bovio).

## Results

### *Dot1l*-cKO^*Atoh1*^ Impairs Granule Cell Development and Function In Vivo

**Fig. 1:**
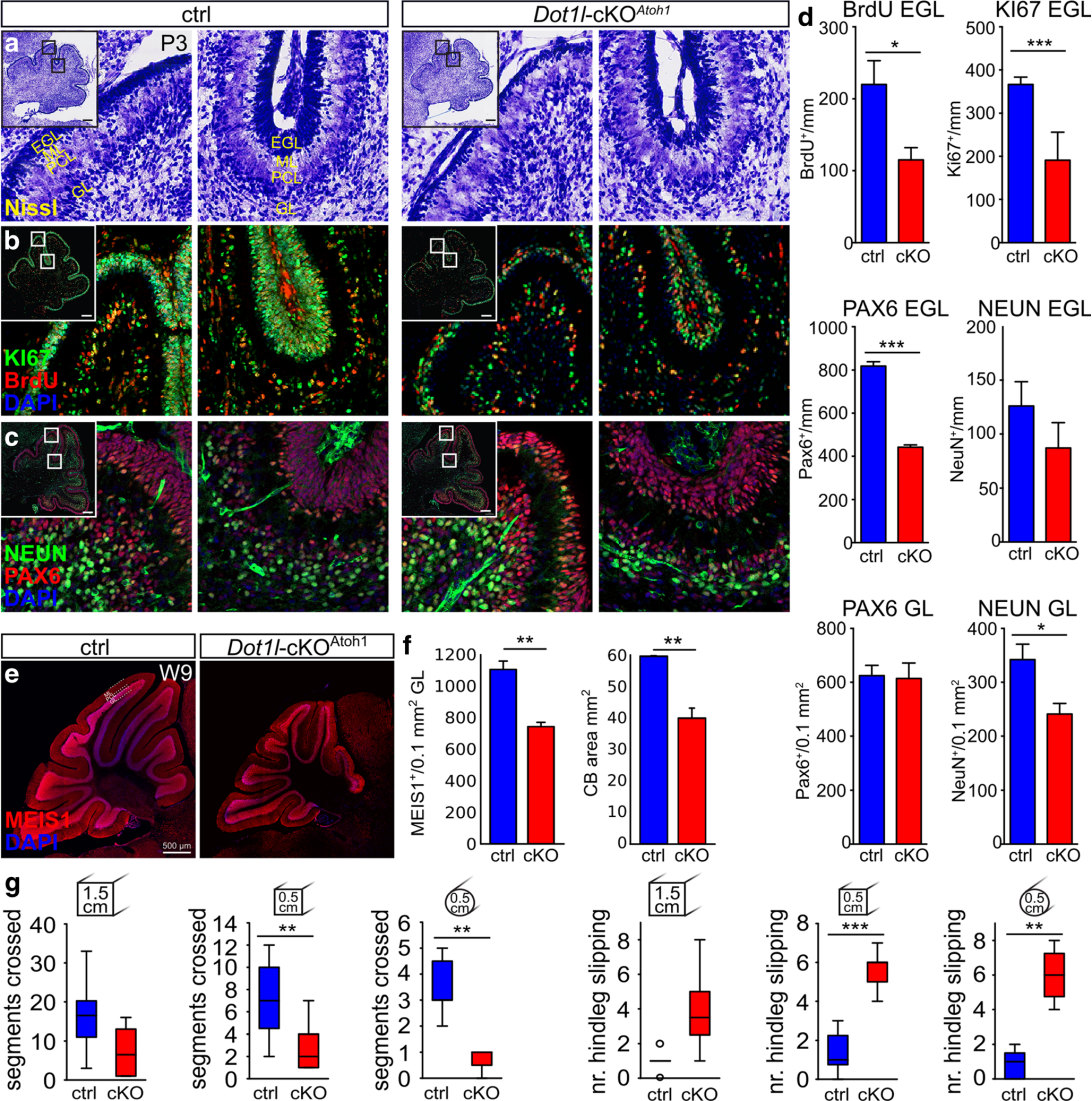
Loss of DOT1L function in vivo in cerebellar granule precursors results in reduced proliferation, fewer differentiated CGN, and impaired motor behavior. **a** Nissl staining of P3 cerebellar sections of control (*Dot1l*^f/f^, *Atoh1*^+/+^) and *Dot1l*-cKO^Atoh1^ reveals a smaller EGL and GL. **b** Immunostaining of P3 cerebellar sections for BrdU and KI67 after a 2 h BrdU pulse, indicating fewer proliferating cells in *Dot1l*-cKO^*Atoh1*^. **c** Immunostaining of P3 cerebellar sections for PAX6 and NeuN, indicating fewer progenitors and reduced number of neurons in *Dot1l*-cKO^*Atoh1*^. Scale bars 200 μm. **d** Quantification of BrdU-, KI67-, PAX6-, and NeuN-positive cells in EGL (per mm length of EGL) and GL (per 0.1 mm^2^ area of GL), respectively. Control (*Dot1l*^f/f^, *Atoh1*^+/+^ and *Dot1l*^f/+^, *Atoh1*^+/+^) *n* = 4 and *Dot1l*-cKO^Atoh1^ (cKO) *n* = 3 as mean ± SEM, two-sided *t* test with unequal variance. **e** Immunostaining of cerebellar sections of 9-week-old mice (W9) for MEIS1 indicates less neurons and a smaller cerebellum upon *Dot1l*-cKO^*Atoh1*^. Scale bar 500 μm. **f** Quantification of MEIS1-positive cells in GL (per 0.1 mm^2^ area of GL) and quantification of the cerebellum in square millimeters. Control (Dot1l^f/f^, Atoh1^+/+^ and Dot1l^f/+^, Atoh1^+/+^) *n* = 3 and *Dot1l*-cKO^Atoh1^ (cKO) *n* = 3 as mean ±SEM, two-sided *t* test with unequal variance. **g** Balance beam motor test with increasing challenge: square 1.5 cm (left), 0.5 cm (middle), and round 0.5 cm (right). Given are the number of segments crossed by the animal (left three panels) and the number of hind leg slipping (right three panels). Nine- to 10-week-old control (*Dot1l*^f/f^, *Atoh1*^+/+^) *n* = 7 and *Dot1l*-cKO^*Atoh1*^*n* = 4 male mice. Data are represented in median whisker-box plots. H0: mutant = ctrl performance, H1: mutant < ctrl performance. One-sided *t* test, unpaired with equal variance. *p ≤ 0.05, **p ≤ 0.005, ***p ≤ 0.0005

To study the impact of DOT1L on the function of the cerebellum in vivo, we conditionally deleted DOT1L in granule cells and PC, respectively. Granule cell progenitors arise in the EGL, migrate to the inner GL, and differentiate to granular neurons. *Atoh1*-cre–expressing mice were used to disrupt *Dot1l* in granule cell precursors (*Dot1l*-cKO^*Atoh1*^). The successful conditional knockout was verified using qRTPCR (Fig. S1A). Immunostaining analysis with antibodies against H3K79me1, H3K79me2, and H3K79me3 suggested less intense fluorescent signal in the EGL for H3K79me1 and H3K79me2 (Fig. S1B). Nissl staining of *Dot1l*-cKO^*Atoh1*^ cerebella revealed a thinner EGL and a slightly disorganized GL (Fig. 1a). BrdU-pulse labelling for 2 h revealed a reduced number of S-phase cells in the EGL of *Dot1l*-cKO^*Atoh1*^ compared to wild-type controls (Fig. 1b, d). We also observed fewer KI67-positive dividing cells as well as PAX6-expressing cells in *Dot1l*-cKO^*Atoh1*^ (Fig. 1b–d). These findings indicated that the EGL of *Dot1l*-cKO^*Atoh1*^ contained less dividing progenitors than controls. Along this line, DOT1L deficiency resulted in fewer differentiated NeuN-expressing neurons compared to wild-type animals in the GL (Fig. 1c, d). NSD2 may also function as histone H3K79 methyltransferase in the cerebellum [37]. However, *Nsd2* transcription did not change upon *Dot1l*-cKO^*Atoh1*^ (Fig. S1C). We observed only few apoptotic activated CASPASE3 (aCASP3)-expressing cells in both mutant and control animals at E18.5, P0, and P3, which excluded cell death as a major cause of the cell loss in the EGL of *Dot1l*-cKO^*Atoh1*^ (Fig. S1D, E).

Adult 9-week-old *Dot1l*-cKO^*Atoh1*^ mice revealed a smaller cerebellum with less MEIS1-positive granular neurons (Fig. 1e, f). In a motor behavior test on a balance beam, DOT1L-deficient mice performed significantly worse than wild-type controls (Fig. 1g). CALB1 immunostainings did not reveal changed numbers or gross morphological alterations of PCs (Fig. S1F).

Using *Pcp2*-cre, we disrupted *Dot1l* in PC (*Dot1l*-cKO^*Pcp2*^). H3K79me1, H3K79me2, and H3K79me3 were still detectable in CALB1-positive cells (Fig. S2A). Morphological and immunohistological analyses of knockout mice compared to those of wild-type mice did not reveal an obvious phenotype (Fig. S2B). In addition, performance of mutant animals in a motor behavior test on a balance beam was comparable to wild-type animals (Fig. S2C), indicating that DOT1L activity might be dispensable for proper function of mature PC. Together this in vivo data delineated that DOT1L function in developing and mature granule cells prevented ataxic behavior.

### In Vivo Targets of DOT1L Affect Cell Migration, Stress Response, Cholesterol Metabolism, and Cell Cycle

**Fig. 2:**
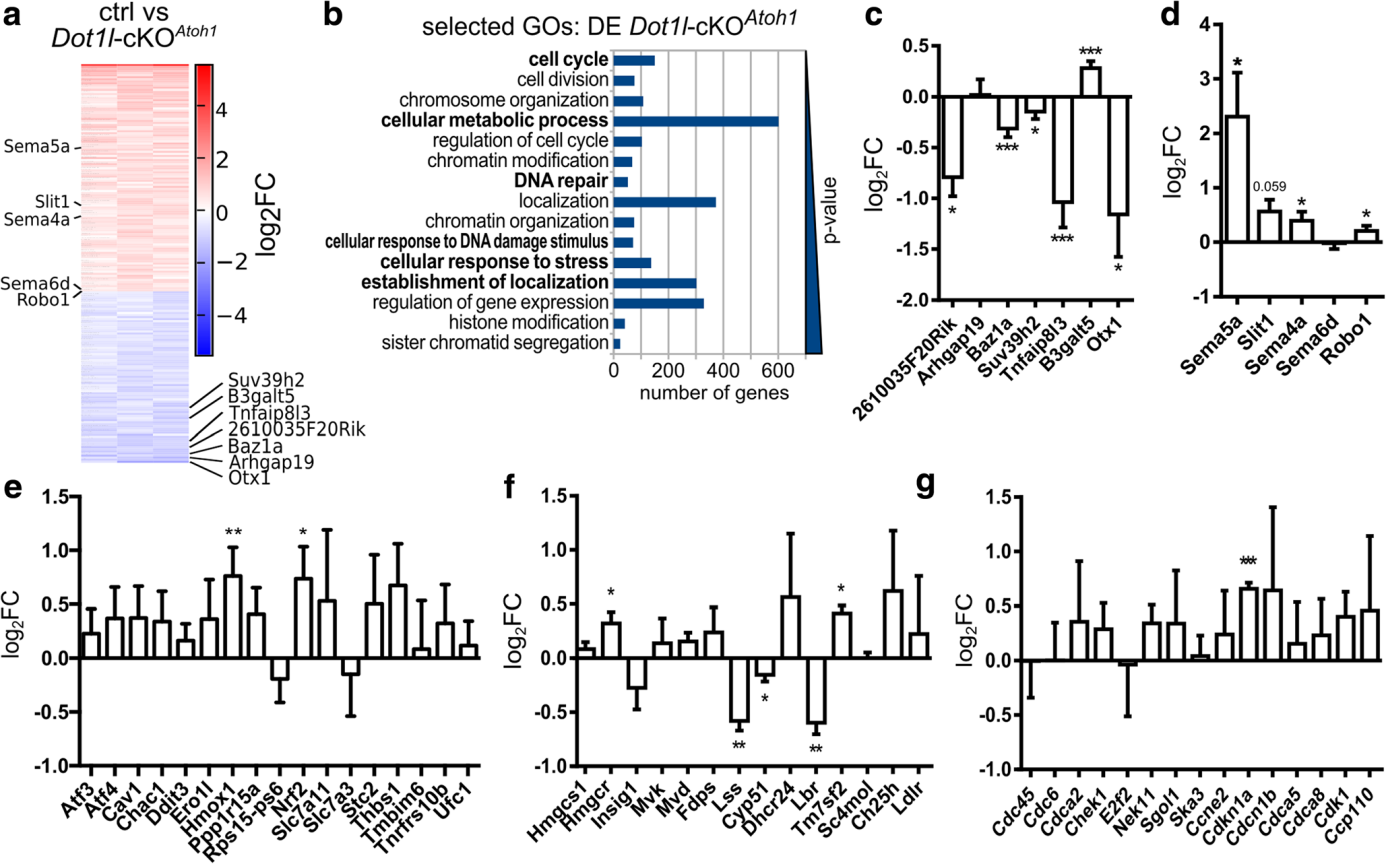
*Dot1l*-cKO^*Atoh1*^ affects expression of genes implicated in axon guidance/cell migration, stress response, cholesterol metabolism, and cell cycle. **a** Heatmap of DE genes as revealed by microarray hybridization (red: increased expression, blue: decreased expression) for *n* = 3 of P3 *Dot1l*-cKO^*Atoh1*^ to control (Dot1l^f/f^, Atoh1^+/+^) mice. FC cut-off ≥ 1.5 and ≤ − 1.5 and *p* ≤ 0.05 displayed as log_2_FC. Selected candidate genes displayed in **c** and **e** are highlighted. **b** Selected enriched GO terms associated with locomo*, migra*, locali*, motil*, cycle*, metabol*, cholest*, lipid*, transport*, stress*, neuro*, cerebell*, projection, axon, and dendri*, within the first 100 most significant terms. Given is the number of genes, ordered from top to bottom according to increasing *p*- values. **c**–**g** qRTPCR validation of DE genes which were revealed by microarrays in **a** displayed in different groups: **c** randomly selected genes from the fraction of top downregulated genes, **d** cell migration, **e** ER stress, **f** lipid and cholesterol metabolism, **g** cell cycle. Given is the log_2_FC ± SEM between P3 *Dot1l*-cKO^*Atoh1*^ and control mice (*n* = 3), two-sided *t* test, with equal variance. *p ≤ 0.05, **p ≤ 0.005, ***p ≤ 0.0005

We next aimed to unravel hitherto unknown target genes of DOT1L in cerebellar granular cells in vivo. We used microarrays to determine the transcriptional changes in *Dot1l*-cKO^*Atoh1*^ compared to wild-type animals. In *Dot1l*-cKO^*Atoh1*^, we found 2236 differentially expressed (DE) genes with a *p*-value ≤ 0.05 (Fig. 2a). As the phenotype of *Dot1l*-cKO^*Atoh1*^ mice suggested that cell proliferation, migration, axon guidance, differentiation, and/or locomotor behavior might be compromised, we subsampled these categories from a GO-term analysis (Fig. 2b). As expected, these analyses revealed DE genes falling in these categories. We selected seven potential target genes with decreased expression levels in *Dot1l*-cKO^*Atoh1*^ and confirmed differential expression in *Dot1l*-cKO^*Atoh1*^ of six of them using qRTPCR on independent samples (Fig. 2c). One of the targets, *B3galt5*, showed however slightly increased expression in qRTPCRs. Next, we assessed candidate genes that increased transcriptionally in *Dot1l*-cKO^*Atoh1*^ and affect cell migration or axon growth/guidance. We tested *Sema5a*, *Slit1*, *Sema4a*, *Sema6d*, and *Robo1* for differential expression in *Dot1l*-cKO^*Atoh1*^ (Fig. 2d) and corroborated that *Sema5a*, *Sema4a*, and *Robo1* expression significantly increased compared to those in controls. Next, we analyzed genes with functions in ER stress. Two ER stress genes, i.e., *Hmox1* and *Nrf2*, were significantly increased in *Dot1l*-cKO^*Atoh1*^ compared to those in controls (Fig. 2e). *Atf3*, *Atf4*, and *Ddit3*, which transcriptionally increased upon in vitro DOT1L inhibition in cortical cells [14], did not reach significant different levels in qRTPCR of *Dot1l*-cKO^*Atoh1*^ cerebella (Fig. 2e). For the cholesterol biosynthesis pathway, we analyzed 14 genes. *Hmgcr*, *Lss*, *Cyp51*, *Lbr*, and *Tm7sf2* transcriptionally changed significantly upon DOT1L deletion in vivo (Fig. 2f). The analysis of cell cycle genes revealed that only *Cdkn1a* significantly increased in vivo upon DOT1L deletion in qRTPCR validation compared to controls (Fig. 2g). With these results, we concluded that *Dot1l*-cKO^*Atoh1*^ in cerebellar granule cells led to significant transcriptional changes of genes which are involved in processes of cell migration, ER stress, cholesterol, and lipid metabolism, as well as cell cycle.

### DOT1L Promotes Proliferation of CGNP In Vitro

**Fig. 3:**
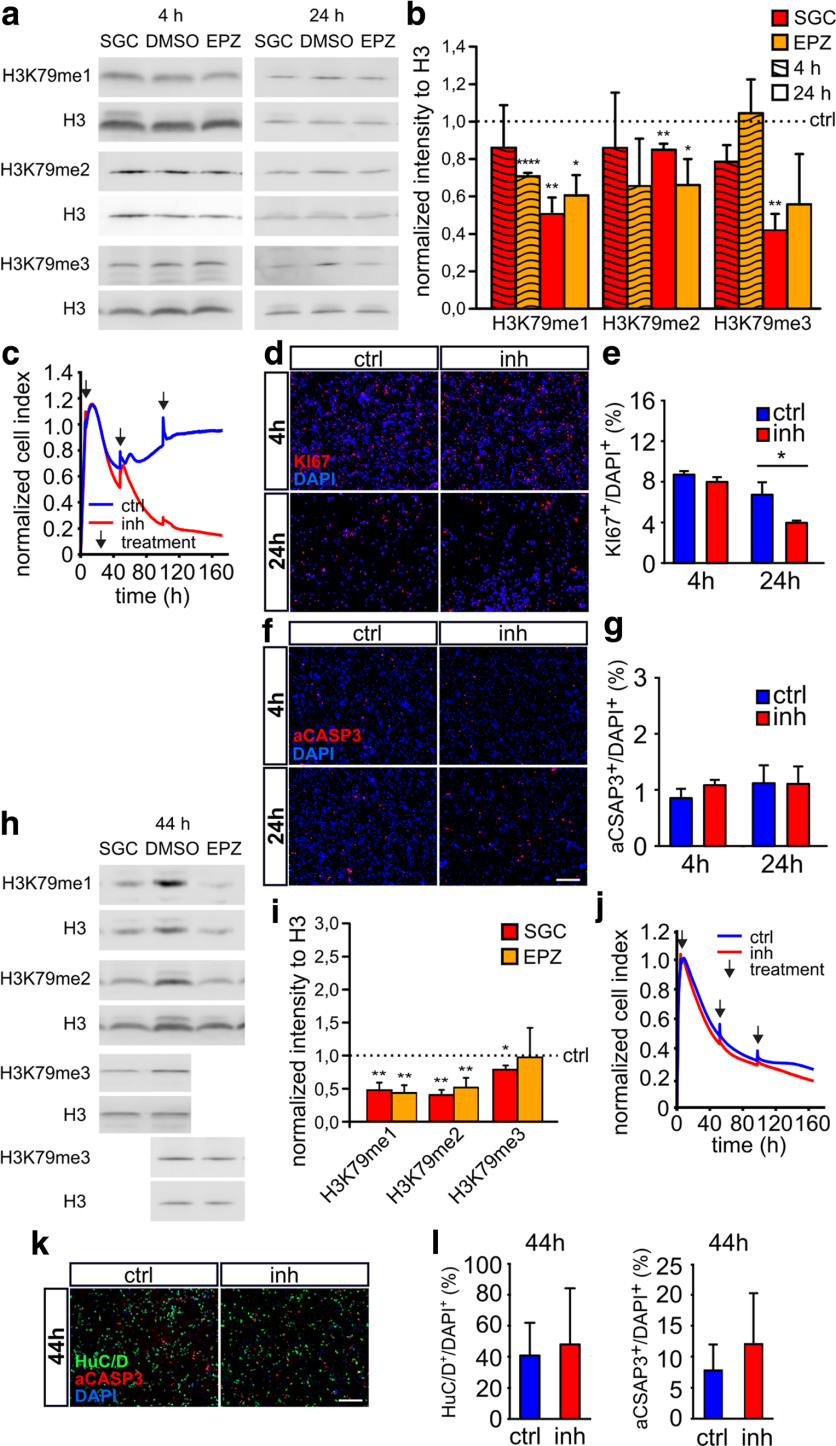
DOT1L activity promotes CGNP proliferation but does not affect CGN survival and differentiation. **a**, **b** Levels of H3K79me1, H3K79me2, and H3K79me3 as shown by immunoblot and densitometric analysis of CGNP after 4 h or 24 h of SGC0946 inhibition (SGC, red bars) or EPZ5676 (EPZ, orange bars) compared to DMSO-treated control (ctrl) represented as dashed horizontal line. *n* = 3, mean ± SEM, two-sided *t* test, equal variance. **c** RTCA shows a reduced normalized cell index upon DOT1L inhibition (red curve, inh) compared to DMSO control (blue, ctrl). One representative experiment out of three is shown. **d**, **e** Immunostainings (ICC) and quantification of KI67-positive CGNP 4 h and 24 h after DOT1L inhibition reveal fewer proliferating cells compared to DMSO-treated controls. Given is the percentage of stained cells per DAPI-positive cells. *n* = 3, mean ± SEM, unpaired two-tailed *t* test with equal variance. **f**, **g** Immunostainings (ICC) and quantification of aCASP3-positive CGNP 4 h and 24 h after DOT1L inhibition or DMSO control. **h**, **i** Immunoblot and densitometric analysis of H3K79me1, H3K79me2, and H3K79me3 after DOT1L inhibition compared to DMSO controls in CGN. Color code as in **b**. *n* = 5, mean ± SEM, two-sided *t* test, equal variance. **j** RTCA of CGN shows no difference between DOT1L inhibition and DMSO treatment. Labelling as in **c**. (**k**, **l**) Immunostainings (ICC) and quantification of HuC/D and aCASP3-positive CGN reveal comparable numbers after DOT1L inhibitor or DMSO treatment. *n* = 5, mean ± SEM, unpaired two-tailed *t* test with equal variance. *p-value ≤ 0.05, ***p value ≤ 0.0005

To analyze DOT1L function in cerebellar granule cells in vitro, we cultivated CGNP and CGN under pharmacological inhibition of DOT1L using two different inhibitors, namely, SGC0946 and EPZ5676. CGNP were treated with DOT1L inhibitors for 4 h or 24 h before they started to differentiate. SGC0946 treatment resulted in fewer H3K79me1, H3K79me2, and H3K79me3 after 24 h but not after 4 h of inhibition as detected by immunoblotting (Fig. 3a, b). Treatment with EPZ5676 resulted in less H3K79me1 after 4 h and 24 h. H3K79me2 and H3K79me3 did not change after 4 h of treatment, but H3K79me2 decreased after 24 h.

Real-time cell analyses (RTCA) of CGNP treated with SGC0946 resulted in a declining relative cell index (Fig. 3c). To analyze whether cell death or reduced cell proliferation caused the declining cell index, we stained fixed CGNP cultures with antibodies against KI67 (Fig. 3d) or aCASP3 (Fig. 3f) after 4 h or 24 h inhibitor treatment with SGC0946. Quantification of the immunostainings revealed that inhibition of DOT1L activity did not result in increased aCASP3-mediated apoptosis but significantly interfered with cell proliferation after 24 h but not after 4 h treatment (Fig. 3e, g). EPZ5676 treatment did not result in significant changes of KI67 after 4 h or 24 h compared to the control condition (Fig. S3A, B).

CGN were treated with DOT1L inhibitor for 44 h during their differentiation process. SGC0946- or EPZ5676-treated CGN had reduced levels of H3K79me1 and H3K79me2 (Fig. 3h, i). SGC0846, but not EPZ5676, treatment led also to reduced H3K79me3. However, reduced H3K79 methylation levels in CGN did not alter the cell index in RTCA compared to DMSO-treated cells in RTCA (Fig. 3j). Likewise, immunostainings for aCASP3 and HuC/D as neuronal marker (Fig. 3k) followed by quantification did not reveal alterations between inhibitor-treated and control cells (Fig. 3l).

In summary, CGNP and CGN cultured in vitro had reduced H3K79 methylation upon DOT1L inhibition with SGC0946 compared to controls. Impaired DOT1L activity led to decreased proliferation and differentiation. These processes seemed also disturbed in *Dot1l*-cKO^*Atoh1*^. We therefore considered in vitro inhibition using SGC0946 as suitable to study DOT1L function on a molecular level.

### Inhibition of DOT1L Activity Alters H3K79me2 in CGNP and CGN

We elucidated how DOT1L activity affected H3K79me2 distribution in cultured CGNP and CGN either treated with DMSO or SGC0946. CGNP were treated for 4 h, as a longer treatment impaired cell proliferation and resulted in massive cell loss (Fig. 3c). CGN were treated for 44 h.

**Fig. 4:**
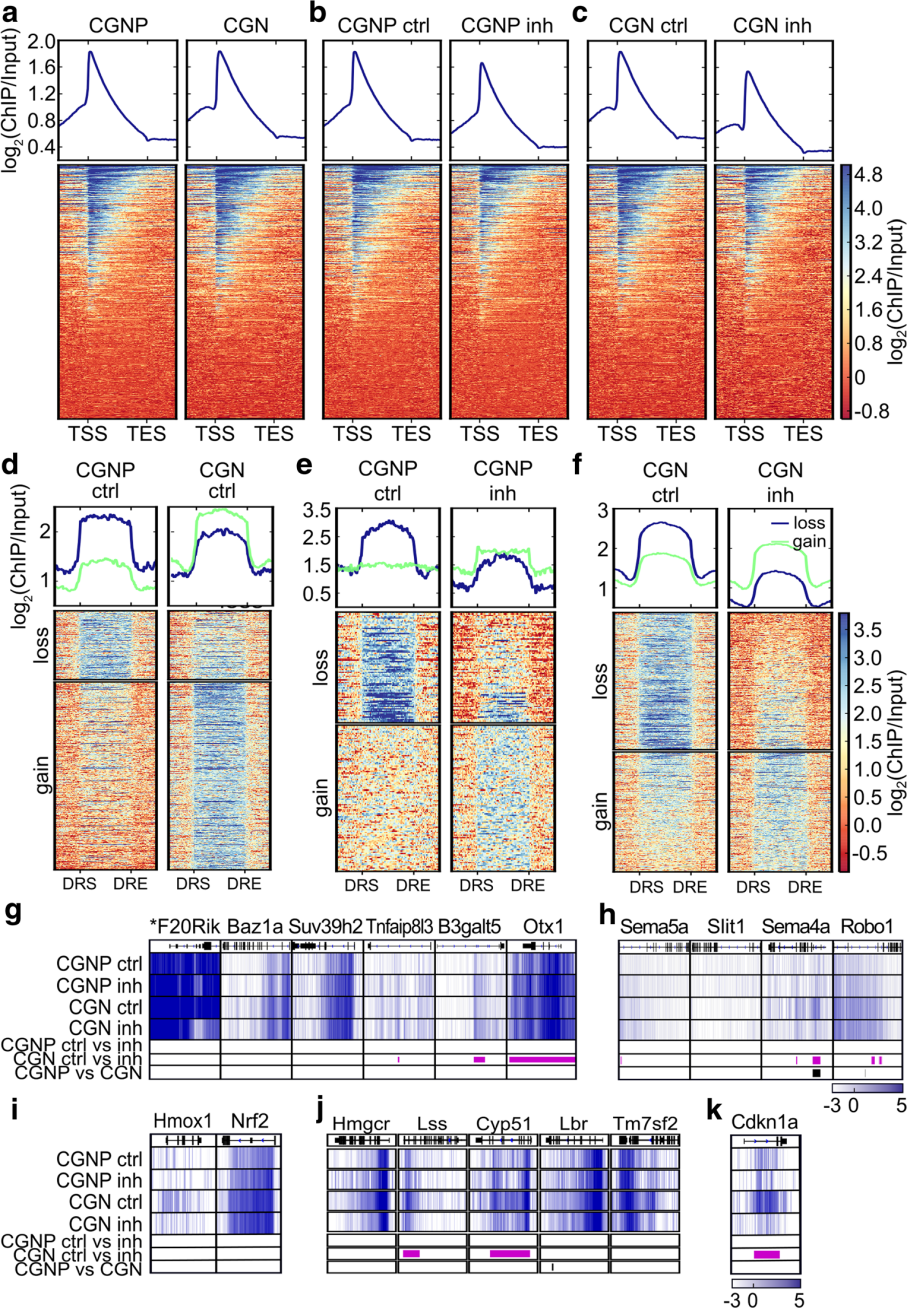
Differential gene expression and H3K79me2 profiles of CGNP and CGN upon DOT1L inhibition and during differentiation. **a**–**c** Genome-wide mean enrichment (top panels) and heatmaps (lower panels) of H3K79me2 in **a** CGNP and CGN during differentiation (DMSO treated) and **b** CGNP and **c** CGN treated with DMSO (ctrl) or SGC0946 (inh) for 4 h (CGNP) or 44 h (CGN) displayed in SES normalized log_2_(ChIP/Input) in a range of 2 kb up- and downstream of the TSS and TES; range from TSS to TES scaled to 4 kb. One representative experiment out of two is shown. **d**–**f** Genome-wide mean enrichment (top panels) and heatmaps (lower panels) of differentially methylated regions (DR) displayed from DRstart (DRS) to DRend (DRE) for *n* = 2, cut-off *q* ≤ 0.1, during differentiation from CGNP to **d** CGN, **e** CGNP, and **f** CGN for DMSO controls and DOT1L inhibition. One representative experiment is displayed in SES normalized log_2_ratio (ChIP/Input), 2 kb up- and downstream of TSS and TES mapped to mm10; TSS to TES scaled to 4 kb. Heatmaps are calculated in two k-mean clusters with reduced (loss) and increased (gain) H3K79me2. **g**–**k** H3K79me2 DR upon DOT1L inhibition of significant DE genes upon *Dot1l*-cKO^*Atoh1*^ displayed as SES normalized log_2_ratio (ChIP/Input). Given are from top to bottom the names of the gene, genomic organization, and DR pattern (white: low methylation, dark blue: high methylation) of DMSO-treated (ctrl) and DOT1L inhibitor–treated (inh) CGNP and CGN, respectively. Significant DR of ctrl- vs. inh-treated samples or during differentiation are indicated as purple or black bars, respectively

First, we compared H3K79me2 occupancy in CGNP to CGN (Fig. 4a) to address developmental dynamics in H3K79me2 distribution. Genome-wide mapping of H3K79me2 did not reveal striking differences of the histone mark in CGNP compared to CGN. Genome-wide mean enrichment of H3K79me2 was highest shortly downstream of the transcriptional start site (TSS) and reduced gradually towards the transcriptional end site (TES). Comparison of the patterns between CGN and CGNP revealed that the regions 5′ to the TSS seemingly increased during development.

Next, we compared H3K79me2 distribution in CGNP or CGN controls with DOT1L-inhibited cells (Fig. 4b, c). DOT1L inhibition in CGNP or CGN affected all regions equally, because the genome-wide mean of H3K79me2 mark appeared generally lower compared to DMSO-treated controls. The shape of the profile did not change.

To analyze at which loci H3K79me2 levels changed between CGNP and CGN during differentiation, we determined differentially methylated regions (DR) (Fig. 4d). In total, we identified 1206 DR regions, of which 323 lost and 883 gained H3K79me2 during differentiation from progenitors to neurons. Thus, H3K79me2 mainly accumulated during maturation of granular neurons.

Acute inhibition of DOT1L in CGNP revealed 195 DR. We hypothesize that the low number of DR resulted from the short-term inhibition (4 h) in CGNP (Fig. 4e), because the longer 44 h treatment of CGNs with DOT1L inhibitor resulted in 8735 DR compared to controls (Fig. 4f). DOT1L inhibition in CGNP led to decreased H3K79me2 enrichment at 84 regions and, surprisingly, to an increased enrichment at 111 regions as compared to the DMSO treatment. In CGN, we observed increasing H3K79me2 levels at 4075 loci and decreased H3K79me2 at 4660 loci upon DOT1L inhibition. DOT1L inhibition resulted in a remarkable decrease of H3K79me2 (Fig. 4e, f), whereas the increase was comparably moderate. Increasing levels of H3K79me2 might indicate incomplete inhibition. Alternatively, increased levels might hint to other H3K79me2 activities than DOT1L, which might be less efficient or acting with slower kinetics. However, the expression levels of *Nsd2* as alternative H3K79me2 methyltransferase did not change upon DOT1L inhibition (Fig. S1C).

GO-term analysis revealed that DR genes between CGNP and CGN and upon DOT1L inhibition were significantly enriched for nervous system development and differentiation, which suggested that H3K79me2 affected gene expression implicated in cerebellar development (Fig. S4A-C).

As shown in Fig. 2c–g, *Dot1l*-cKO^*Atoh1*^ led to verified, significant transcriptional change of 18 genes affecting cell migration, ER stress, cholesterol metabolism, and cell cycle. To elucidate whether expression changes of these genes correlated with altered levels of H3K79me2 in vitro, we compared H3K79me2 distribution after DOT1L inhibition either in (1) CGNP or in (2) CGN and (3) during development from CGNP to CGN without DOT1L inhibition.

We did not observe any changes after short-term inhibition in CGNP for any of the 18 target genes. For nine genes, i.e., *Tnfaip8l3*, *B3galt5*, *Otx1*, *Sema5a*, *Sema4a*, *Robo1*, *Lss*, *Cyp51*, and *Cdkn1a*, H3K79me2 levels changed upon DOT1L inhibition in cultured CGN, indicating a direct DOT1L effect (Fig. 4g–k). For two of these genes, i.e., *Sema4a* and *Lbr*, H3K79me2 levels increased during CGNP-CGN differentiation (Fig. 4h, j), indicating a developmental accumulation of H3K79me2. Taken together, we identified nine putative direct DOT1L target genes in CGN, which are implicated in signaling (*Tnfaip8l3*, *B3galt5*), in transcriptional regulation (*Otx1*), in cell migration (*Sema5a*, *Sema4a*, *Robo1*), in cholesterol metabolism (*Lss, Cyp51*), and in cell cycle (*Cdkn1a*) (summarized in supplementary in Table S1).

### Inhibition of DOT1L Activity Alters the Transcriptome in CGNP and CGN

**Fig. 5:**
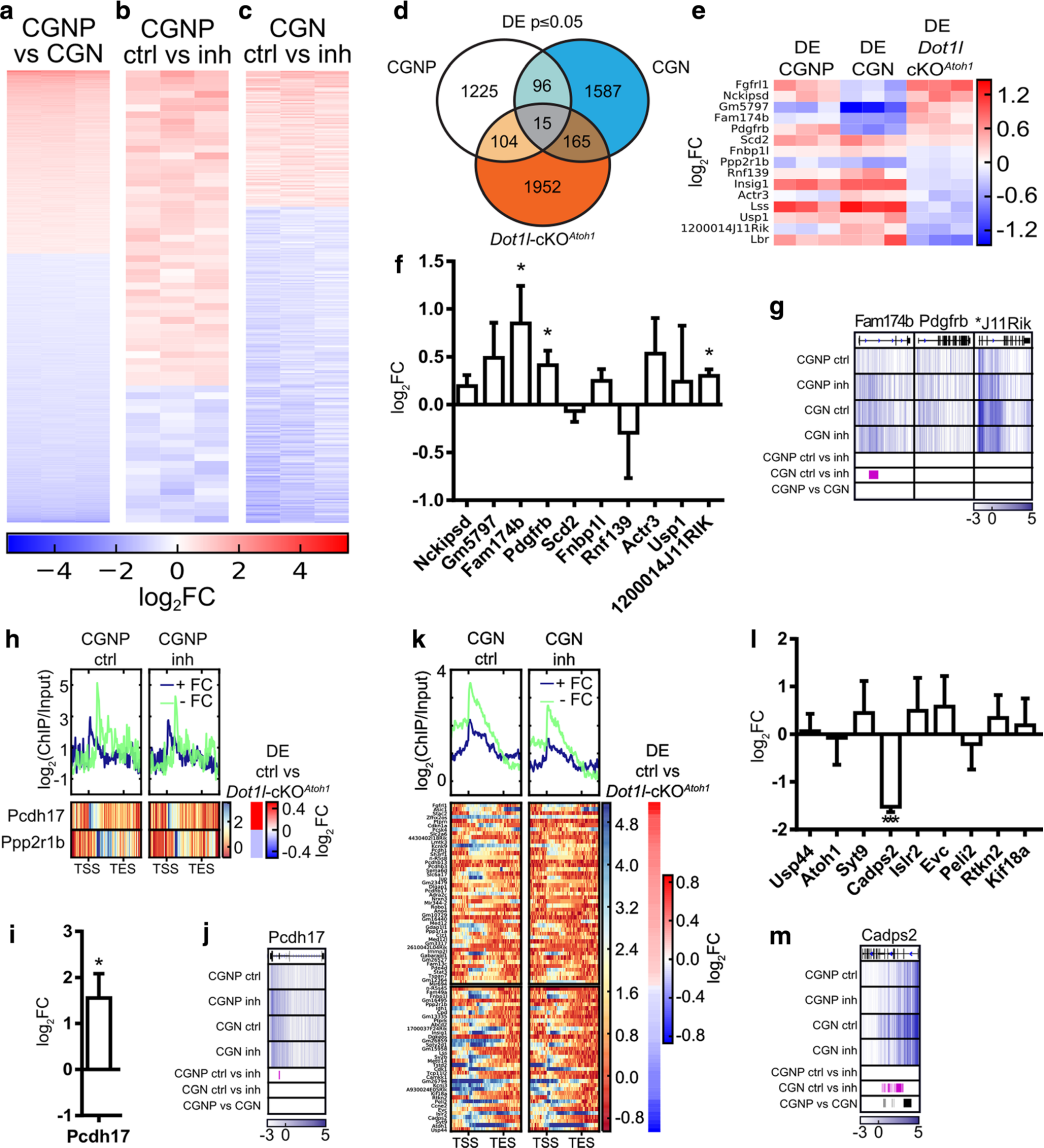
Determination of DOT1L in vivo target genes by comparison to candidate genes resulting from in vitro DE and DE/DR genes upon DOT1L inhibition. **a**–**c** Heatmaps of DE genes with a fold change (FC) cut-off ≥ 1.5 and ≤ − 1.5 and *p*-value ≤ 0.05 on a log_2_FC scale; red increased expression, blue decreased expression. DE genes during **a** CGNP to CGN differentiation, **b** CGNP after 4 h of DOT1L inhibition, and **c** CGN 44 h after DOT1 inhibition, compared to DMSO-treated controls. **d** Venn diagram of DE genes in CGNP (ctrl vs. inh treated), CGN (ctrl vs. inh treated), and wt vs. *Dot1l*-cKO^*Atoh1*^ mice. Cut-off for DE genes *p* ≤ 0.05. **e** Heatmap of 15 genes shared with all three datasets sorted according to transcriptional increase (red) or decrease (blue) in *Dot1l*-cKO^*Atoh1*^. **f**, **i**, **l** qRTPCR of candidate genes in P3 wt vs. *Dot1l*-cKO^*Atoh1*^. Mean log_2_FC ± SEM, *n* = 3, two-sided *t* test with equal variance. *p ≤ 0.05, **p ≤ 0.005, ***p ≤ 0.0005. **g**, **j**, **m** Overlay of H3K79me2 (*n* = 2) in SES normalized log_2_ratio (ChIP/Input) and DR, cut-off *q* ≤ 0.1 each, is displayed. Given are from top to bottom the names of the gene, genomic organization, and DR pattern (white: low methylation, dark blue: high methylation) of DMSO-treated (ctrl) and DOT1L inhibitor–treated (inh) CGNP and CGN, respectively; significant DR regions of ctrl- vs. inh-treated samples or during differentiation indicated as purple or black bars, respectively. **h**, **k** Mean enrichment (top panels) and heatmaps (lower panels) for DE and DR genes, cut-off *p* ≤ 0.05, in **h** CGNP and in **k** CGN upon DOT1L inhibition and in DMSO-treated controls. Gene order according to increased (red) or decreased (blue) expression in *Dot1l*-cKO^Atoh1^ vs. controls, given in a log_2_FC. **f** qRTPCR and **g** genome track corresponding to **e** intersecting heatmap of DE genes. **i** qRTPCR and **j** genome track corresponding to **h** heatmap of DR in DOT1L inhibitor–treated CGNP compared to control. **l** qRTPCR and **m** genome track corresponding to **k** heatmap of DR in DOT1L inhibitor–treated CGN compared to control

We further characterized DOT1L function in in vitro cultured CGNP and CGN using microarrays to analyze the transcriptomes of CGNP and CGN during differentiation and after DOT1L inhibitor treatment. Differentiation from CGNP to CGN led to 9148 DE genes (Fig. 5a). DOT1L inhibition led to 1440 DE genes in CGNP (Fig. 5b) and 1863 DE genes in CGN (Fig. 5c). We observed increased and decreased transcription upon inhibited DOT1L activity compared to the control condition. Among significantly enriched GO terms of in vitro DE genes after DOT1L inhibition, we identified cell migration, stress response, cholesterol and lipid metabolism, and cell cycle (Fig. S5A–C). These terms were also enriched among DE genes of *Dot1l*-cKO^*Atoh1*^ (Fig. 2b).

We next assessed whether we could use DE/DR correlations revealed from in vitro cultivated cells to identify systematically direct DOT1L targets that would also transcriptionally change in vivo upon DOT1L deletion. At first, we correlated alterations of the H3K79me2 pattern within the fractions of DE genes of cultivated CGNP and CGN. During CGNP to CGN differentiation, 347 out of 9148 DE genes changed H3K79me2 levels (Fig. S5D). Twelve genes out of 1440 DE genes in CGNP treated with DOT1L inhibitor had altered H3K79me2 levels (Fig. S5E). From 1863 DE genes of CGN treated with DOT1L inhibitor, 432 genes showed a changed H3K79me2 level compared to DMSO-treated cells (Fig. S5F). Generally, H3K79me2 levels were reduced after SGC0946 inhibitor treatment in CGNP and CGN (Fig. S5E–F). However, decreased H3K79me2 after DOT1L inhibition compared to DMSO treatment was associated with both, increasing and decreasing expression levels. Thus, inhibition of DOT1L led to reduced or increased transcription of genes, and genes of both categories were marked with H3K79me2.

In a second step, we intersected the DE genes that we revealed by (1) pharmacological DOT1L inhibition of CGNP and CGN in vitro and (2) through *Atoh1*-Cre–driven DOT1L deletion in vivo (Fig. 5d). In all, we identified only 15 significant DE genes in the intersection of the three model systems (Fig. 5d, e). Thirteen of these genes were subjected to qRTPCR validation and revealed that *Fam174b*, *Pdgfrb*, *1200014J11Rik* (Fig. 5f), *Lss*, and *Lbr* (Fig. 2f) were significantly altered. Only *Fam174b* and *Lss* were also DR upon DOT1L inhibition in vitro (Figs. 4j and 5g), a finding that rendered transcription of these two targets as H3K79me2 dependent. The small fraction of 15 overlapping DE genes together with the finding that only a minor fraction out of these was significantly altered in vivo suggested that the *Dot1l*-cKO^*Atoh1*^ phenotype might be caused by altered gene transcription of CGNP- or CGN-specific genes and not by genes that are expressed in both cell types. This assumption was corroborated by a larger overlap between the fractions of CGNP or CGN DE genes, respectively, with the *Dot1l*-cKO^*Atoh1*^ transcriptome. Here, CGNP shared 104 DE genes with *Dot1l*-cKO^*Atoh1*^, whereas 165 genes overlapped between CGN and *Dot1l*-cKO^*Atoh1*^ (Fig. 5d, Fig. S5G–I).

Among the 104 DE genes shared between CGNP and *Dot1l*-cKO^*Atoh1*^, only two were also DR (Fig. 5h, j). We confirmed successfully that expression of *Pcdh17* significantly increased in *Dot1l*-cKO^*Atoh1*^ compared to that in control animals in vivo (Fig. 5i), but we experimentally failed to amplify specifically *Ppp2r1b*. For CGN, we revealed a higher fraction of in total 80 genes which were DE in vitro as well as in vivo and were in addition DR. Out of these candidates that were all DR and therefore most likely direct target genes of DOT1L and H3K79me2 (Fig. 5k), we chose a subset of nine genes with known functional roles in cerebellar function for validation. However, qRTPCRs revealed that only *Cadps2* expression decreased significantly in transcription after DOT1L deletion (Fig. 5l). *Cadps2* gained H3K79me2 during CGNP-CGN differentiation and lost H3K79me2 upon DOT1L inhibition in CGN (Fig. 5m).

In summary, the attempt to identify direct DOT1L target genes by combining transcriptomic data from in vitro and in vivo model systems of impaired DOT1L function revealed three genes (*Fam174b*, *Pcdh17*, *Cadps2*). Together with the nine direct targets that we identified starting our analysis with in vivo DE genes and correlating the DR pattern in vitro (Figs. 2 and 4), our study revealed 12 direct DOT1L target genes. Although transcription of these 12 genes significantly changed in *Dot1l*-cKO^Atoh1^, their expression pattern after DOT1L inhibition in vitro varied substantially (Fig. S6A–C). Only *B3galt5* and *Pcdh17* increased in vitro in presence of both inhibitors in CGN and recapitulated the in vivo observations.

Taken together, our study identified potential direct in vivo targets of DOT1L in the cerebellum, which are implicated in diverse processes such as cell migration and axon growth/guidance (*Sema4a*, *Sema5a*, *Robo1*), cholesterol and lipid metabolism (*Lss*, *Cyp51*), signaling (*Tnfaip8l3*, *B3galt5*), transcription (*Otx1*), cell cycle (*Cdkn1a*), calcium-dependent cell adhesion or exocytosis (*Pcdh17*, *Cadps2*), and unclassified functions (*Fam174b*). Dysregulation of expression of these candidates and/or processes might be implicated in the ataxia phenotype of *Dot1l*-cKO^*Atoh1*^.

## Discussion

### *Dot1l*-cKO^*Atoh1*^ Leads to Ataxia in Mice

The results presented in this study show that DOT1L activity is necessary for proper function of the cerebellum. Behavioral tests of mice with granule cell-specific DOT1L deletion in the cerebellum indicated a mild ataxia phenotype. DOT1L deficiency impaired granule cell proliferation and neuronal differentiation during cerebellar development. However, DOT1L function might be dispensable for motor function of PC as *Dot1l*-cKO^*Pcp2*^ mice performed equally well as controls in the applied behavioral tests. However, *Pcp2*-Cre is active in mature PC only [19]. Thus, we cannot exclude that DOT1L is important for PC progenitor cells and/or their differentiation into mature neurons.

### H3K79 Methylation Can Activate or Repress Transcription

To reveal specific DOT1L target genes in CGNP or CGN, we extensively characterized in vitro cultured CGNP and CGN under pharmacological inhibition of DOT1L by exploring transcriptional changes and alterations of the H3K79me2 pattern. CGNP proliferation was impaired upon DOT1L inhibition verifying the proliferation defect detected for granule cells in *Dot1l*-cKO^*Atoh1*^. In contrast, in CGN, we did not detect functional consequences after DOT1L inhibitor treatment. But further in-depth studies might be needed to uncover potential functions of DOT1L in CGN. To determine direct DOT1L target genes, we analyzed H3K79me2 levels via ChIP-seq of CGNP and CGN treated with DOT1L inhibitor. With that, we could identify differentially methylated regions. In total, our attempt suggested 12 DE genes upon *Dot1l*-cKO^*Atoh1*^ that classified as direct targets. This very low number of retrieved genes indicated to us that the combination of in vivo and in vitro model systems might be of limited use to identify high numbers of relevant direct targets. However, DOT1L inhibitor treatment led to less H3K79me1, H3K79me2, and H3K79me3 in some of the experimental conditions. We solely addressed changes in H3K79me2 in this study. Therefore, we cannot rule out that alterations of H3K79me1 and H3K79me3 would be better suited to identify in vitro DR genes with relevant expression changes under in vivo *Dot1l*-cKO conditions. It is of note that DOT1L might have other additional functions that are independent of H3K79 methylation. Inhibition of DOT1L in CGNP for 4 h led to 104 DE genes, but only two of them were also DR. Although we cannot rule out that all transcriptional changes were induced by the two DR genes in a secondary event, it is also possible that DOT1L affected transcription in an H3K79 methylation-independent manner.

It is equally difficult to predict whether altered H3K79me2 levels correlate with activation or repression of transcription. This notion is for example corroborated by Fig. 4f. In this experiment, we plotted DE and DR genes and we unraveled that both increased and decreased transcriptions coincided with fewer H3K79me2 in CGN treated with the DOT1L inhibitor SGC0946. This shows that H3K79me2 might be interpreted in different ways with regard to transcription and emphasizes that in vivo and in vitro data need to be carefully analyzed and compared.

### DOT1L Direct Targets Influence Essential Processes in Cerebellar Granule Cells

Despite the low numbers of direct targets genes revealed in this study, the identified targets might be relevant for proper cerebellar function and implicated in the mild ataxia phenotype of *Dot1l*-cKO^*Atoh1*^. Axon guidance cues, i.e., *Sema5a*, *Sema4a*, and *Robo1* expression, increased in *Dot1l*-cKO^*Atoh1*^. Loss-of-function experiments showed that semaphorins and ROBO1 influence neurite outgrowth in neurons [38, 39]. The consequences of excessive expression of axon guidance cues are, however, less well described. But interestingly, it was reported that axonal growth cones from cultured hippocampal neurons collapse if exposed to SEMA4A [40]. Excessive expression of *Sem4a* in *Dot1l*-cKO^*Atoh1*^ might therefore impair neuronal differentiation and function.

DE genes of the *Dot1l*-cKO^*Atoh1*^ were enriched in GO-term categories associated with the response to stress. However, in contrast to cerebral progenitors, in which *Atf3*, *Atf4*, and *Ddit3* increased significantly under pharmacological inhibition of DOT1L [14], this activation of the ER stress pathway was not observed in *Dot1l*-cKO^*Atoh1*^in vivo.

DOT1L function affected expression of genes implicated in cholesterol synthesis pathway. Cholesterol is an important molecule for brain development and function, and proper homeostasis is a prerequisite for membrane functions. Endogenous synthesis of cholesterol seemingly occurs in all CNS cell types including neurons [41]. Disturbance of the cholesterol balance in the brain results in neuronal degeneration, synaptic malfunctions, or impaired neurotransmission. Several neurological pathologies are therefore associated with disturbed cholesterol balance [42, 43], and cholesterol seems also involved in Alzheimer’s [44] or Huntington’s disease [45]. Transcription of *Lss* and *Cyp51* decreased in *Dot1l*-cKO^*Atoh1*^ compared to that in control animals. Both enzymes affect the post-squalene part of the cholesterol biosynthesis pathway. Further biochemical analysis might be needed to address whether reduced expression of these post-squalene enzymes would result in ataxia.

DOT1L activity affects cell cycle progression in various organ systems [14, 46, 47]. However, the set of candidate genes that are transcriptionally altered upon loss or inhibition of DOT1L seems to vary within the different cell types. Here, we identified in *Dot1l*-cKO^*Atoh1*^ significantly increased transcription of *Cdkn1a* compared to controls*.* In cortical cells however, *Ccnd1*, *Vangl2*, and *Cenpj* are targets of DOT1L (Franz et al., accepted manuscript, “DOT1L promotes progenitor proliferation and primes for neuronal layer identity in the developing cerebral cortex”, Nucleic Acid Research, 2018). CDKN1A stops proliferation in G1 phase, and its increased expression in *Dot1l*-cKO^*Atoh1*^ correspond to the observation of reduced numbers of progenitors in the EGL.

We further identified *Fam174b*, *Pcdh17*, *Cadps2*, *Tnfaip8l3*, *B3galt*, and *Otx1* as direct target of DOT1L. Whereas no specific functions of *Fam174b*, *Tnfaip8l3*, and *B3galt* are assigned to cerebellar functions as yet, the other candidates putatively contribute to the observed histological and functional phenotypes of *Dot1l*-cKO^Atoh1^. PCDH17 promotes organization of neuronal circuits in different neuroanatomical locations [48, 49]. Human patients presenting with mood disorders have increased transcript levels of *PCDH17*. In vitro, overexpression of PCDH17 in cortical neurons results in synaptic alterations including decreased spine density and abnormal dendritic morphology [50]. It is therefore likely that the increased level of *Pcdh17* transcription in *Dot1l*-cKO^Atoh1^ resulted in synaptic impairment involved in the ataxia observed in these mice.

*Cadps2* was a DOT1L-dependent gene and its expression levels need to be tightly controlled, as imbalanced levels are associated with neurological diseases such as autism [51, 52]. Decreased levels of *Cadps2* in the cerebellum affect neuronal morphology, synapse function, and thus locomotor behavior [51]. *Dot1l*-cKO^*Atoh1*^ express reduced levels of *Cadps2*, which is therefore another likely candidate implicated in the impaired locomotor behavior observed in our study.

*Otx1* was one of the few transcription factors within the cerebellum that depends on DOT1L function. *Otx1* is implicated in development of the cerebellum and the cerebral cortex in mediating specific cell identities [51, 53]. It is therefore a potentially important target of DOT1L to secure proper development and neuronal function of the cerebellum.

In summary, our data showed that DOT1L function is needed for proper development and function of the cerebellum and that impaired DOT1L function results in ataxia in vivo. We identified a small number of candidate genes regulated by DOT1L in vivo and in vitro, misexpression of which might result in ataxia.

## Electronic Supplementary Material

Supplementary Figure 1 H3K79me1 and H3K79me2 are reduced; apoptosis and Purkinje cells are not impaired upon *Dot1l*-cKO^*Atoh1*^. (A) qRTPCR of *Dot1l* exon 2 in P3 wt vs. *Dot1l*-cKO^*Atoh1*^. Mean log_2_FC ± SEM, *n* = 6, two-sided t-test with equal variance. ***: *p* ≤ 0.0005 (B) Immunostaining (IHC) of H3K79me1, H3K79me2 and H3K79me3 of P3 control (*Dot1l*^f/f^, *Atoh1*^+/+^) and *Dot1l*-cKO^*Atoh1*^ cerebellum. Scale bars: 200 μm or 50 μm respectively. (C) qRTPCR of *Nsd2* in P3 control vs. *Dot1l*-cKO^*Atoh1*^. Mean log_2_FC ± SEM, *n* = 3. (D) Immunostaining and quantification of aCASP3 of P3 control (*Dot1l*^f/f^, *Atoh1*^+/+^) and *Dot1l*-cKO^*Atoh1*^ cerebellum. Scale bar: 200 μm. (E) Immunostaining of CALB1 and MEIS1 of cerebelli from 9 week (W) old control (*Dot1l*^f/f^, *Atoh1*^+/+^) and *Dot1l*-cKO^*Atoh1*^, Scale bars: overview 500 μm, right images maximal projection of confocal imaging 50 μm. EGL: external granular layer, ML: molecular layer, PCL: Purkinje cell layer, GL: granular layer. (PNG 2984 kb)

Supplementary Figure 2 Conditional inactivation of DOT1L does not affect Purkinje cell development in *Dot1l*-cKO^*Pcp2*^. (A) Immunostaining of H3K79me1, H3K79me2 and H3K79me3 of P3 control (*Dot1l*^f/f^, *Pcp2*^+/+^) and *Dot1l*-cKO^*Pcp2*^ cerebellum. Scale bars: 200 μm or 50 μm respectively. (B) Nissl stainings and immunostainings of CALB2 and NeuN of P7 old control (*Dot1l*^f/f^, *Pcp2*^+/+^) and *Dot1l*-cKO^*Pcp2*^ mice do not show phenotypic differences. Scale bar: 200 μm. (C) Balance beam motor test with increasing challenge through decreased beam diameters and form: square 1.5 cm (left), 0.5 cm (middle) and round 0.5 cm (right). Control (*Dot1l*^f/f^, *Pcp2*^+/+^) *n* = 8 and *Dot1l*-cKO^*Pcp2*^*n* = 5, 9 to 10 W old male mice. Given is the number of segments crossed and of hind leg slipping. Data represented in median whisker-box plots. H0: mutant = ctrl performance, H1: mutant < ctrl performance; one-sided t-test, unpaired, equal variance. H0 cannot be rejected. (D) Schematic drawing of the experimental set-up of the balance beam motor test. (PNG 7973 kb)

Supplementary Figure 3 Proliferation of CGNP is not impaired upon DOT1L inhibition with EPZ5676. (A) Immunostaining (ICC) and (B) quantification of KI67 positive CGNP 4 h and 24 h after DOT1L inhibition with EPZ5676 (red bars) and DMSO control (blue bars). Given is the percentage of stained cells per DAPI positive cells. n = 3, mean ± SEM, unpaired two-tailed t-test with equal variance. (PNG 405 kb)

Supplementary Figure 4 GO term analysis for biological processes of differentially methylated (DR) genes. (A-C): Selected GO terms associated to locomo*, migra*, locali*, motil*, cycle*, metabol*, cholest*, lipid*, transport*, stress*, neuro*, cerebell*, projection, axon, dendri* and related terms. First among the 100 most significant terms were preferentially selected. Arrangement from top to bottom according to increasing *p*-value. Bars represent the number of genes falling in each GO term category. (PNG 377 kb)

Supplementary Figure 5 Analysis of differentially expressed genes (DE) in CGNP and CGN upon DOT1L inhibition. (A-C): Selected GO terms associated to locomo*, migra*, locali*, motil*, cycle*, metabol*, cholest*, lipid*, transport*, stress*, neuro*, cerebell*, projection, axon, dendri* and related terms. First among the 100 most significant terms were preferentially selected. Arrangement from top to bottom according to increasing *p*-value. Bars represent the number of genes falling in each GO term category. (D-F) Mean enrichment (top panels) and heatmaps (lower panels) for DE genes (*p*-value cut-off: *p* ≤ 0.05, n = 3) that are as well differentially methylated for H3K79me2 (DE/DR genes). Heatmaps were separated in increased (red) or decreased (blue) differential expression, and sorted after DE level represented as log_2_FC. (D) For differentiation from CGNP to CGN, (E) CGNP treated with DOT1L inhibitor for 4 h and (F) CGN treated with DOT1L inhibitor for 44 h. (G) Comparison of DE genes in CGNP ctrl vs. inhibitor treatment with DE genes of CGN ctrl vs. inhibitor. (H) Comparison of DE genes in CGNP ctrl vs. inhibitor treatment with DE genes of *Dot1l*-cKO^*Atoh1*^ vs. ctrl cerebellum. (I) Comparison of DE genes in CGN ctrl vs. inhibitor treatment with DE genes of *Dot1l*-cKO^*Atoh1*^ vs. ctrl cerebellum. Cut-off *p*-value ≤0.05, displayed in log_2_FC, red: increased expression, blue: decreased expression. (PNG 1965 kb)

Supplementary Figure 6 Verification of 12 DOT1L target genes in CGNP and CGN cells treated with inhibitor SGC0946 (SGC) or EPZ5676 (EPZ). (A-C) qRTPCR of putative DOT1L target genes in CGNP treated with DOT1L inhibitors SGC (red bars) and EPZ (orange bars) for 4 h (A) or 24 h (B), and CGN treated with DOT1L inhibitors for 44 h (C). Given is the log_2_FC ± SEM between SGC0946 or EPZ5676 and controls (n = 3), two-sided t-test, with equal variance. *: p ≤ 0.05, **: *p* ≤ 0.005, ***: *p* ≤ 0.0005. (PNG 320 kb)

Supplementary Table S1 Putative direct DOT1L target genes in the cerebella granule cells. (PDF 25 kb)
