# Supplementary figures and images for: Differential Methylation of H3K79 Reveals DOT1L Target Genes and Function in the Cerebellum In Vivo
Source: Mol Neurobiol. 2018 Oct 10;56(6):4273–87. doi: 10.1007/s12035-018-1377-1 (PMC6505521; doi:10.1007/s12035-018-1377-1)

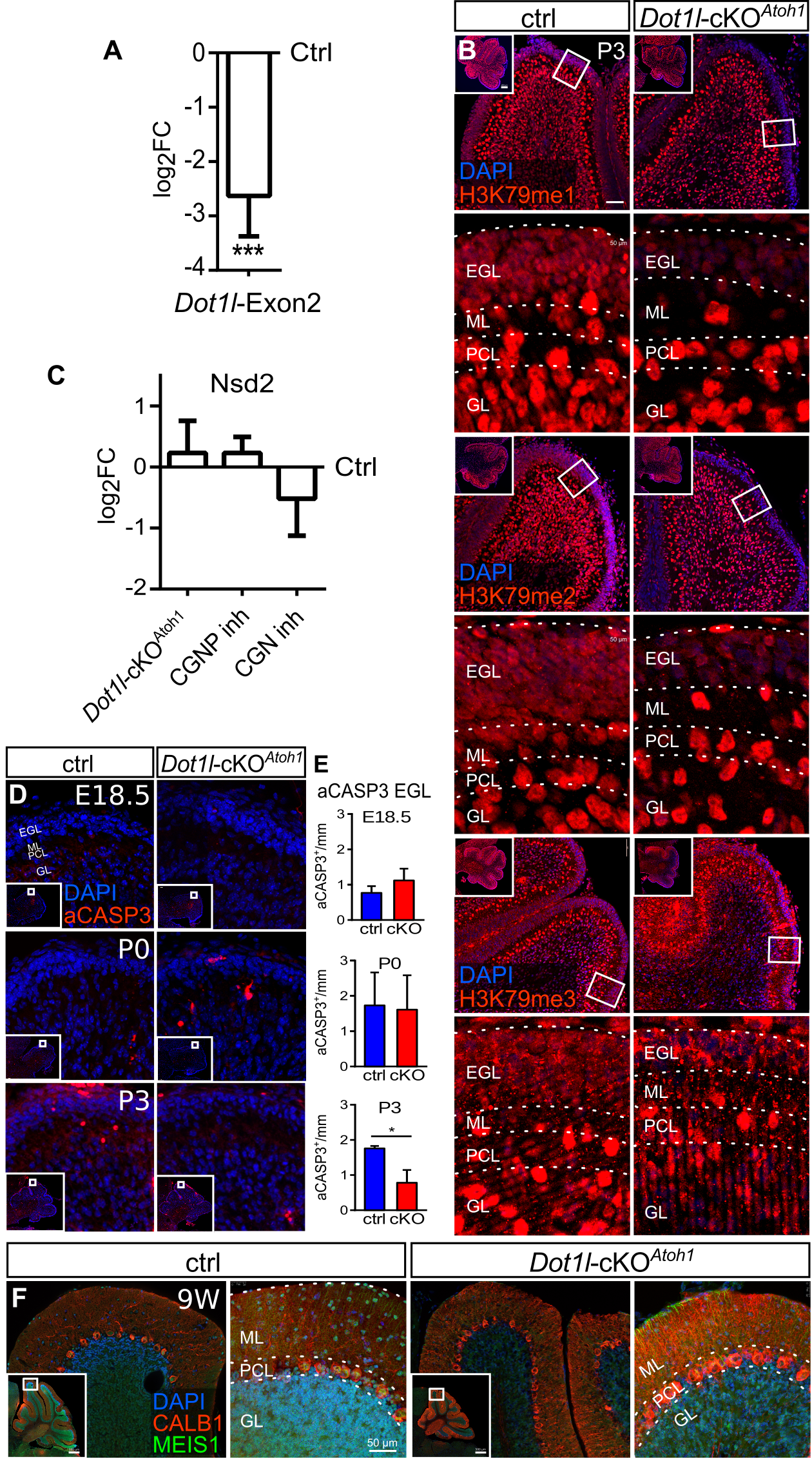

Supplement: Supplementary file 1 — H3K79me1 and H3K79me2 are reduced; apoptosis and Purkinje cells are not impaired upon Dot1l-cKOAtoh1. (A) qRTPCR of Dot1l exon 2 in P3 wt vs. Dot1l-cKOAtoh1. Mean log2FC ± SEM, n = 6, two-sided t-test with equal variance. ***: p ≤ 0.0005 (B) Immunostaining (IHC) of H3K79me1, H3K79me2 and H3K79me3 of P3 control (Dot1lf/f, Atoh1+/+) and Dot1l-cKOAtoh1 cerebellum. Scale bars: 200 μm or 50 μm respectively. (C) qRTPCR of Nsd2 in P3 control vs. Dot1l-cKOAtoh1. Mean log2FC ± SEM, n = 3. (D) Immunostaining and quantification of aCASP3 of P3 control (Dot1lf/f, Atoh1+/+) and Dot1l-cKOAtoh1 cerebellum. Scale bar: 200 μm. (E) Immunostaining of CALB1 and MEIS1 of cerebelli from 9 week (W) old control (Dot1lf/f, Atoh1+/+) and Dot1l-cKOAtoh1, Scale bars: overview 500 μm, right images maximal projection of confocal imaging 50 μm. EGL: external granular layer, ML: molecular layer, PCL: Purkinje cell layer, GL: granular layer. (PNG 2984 kb) [file 12035_2018_1377_MOESM1_ESM.png]

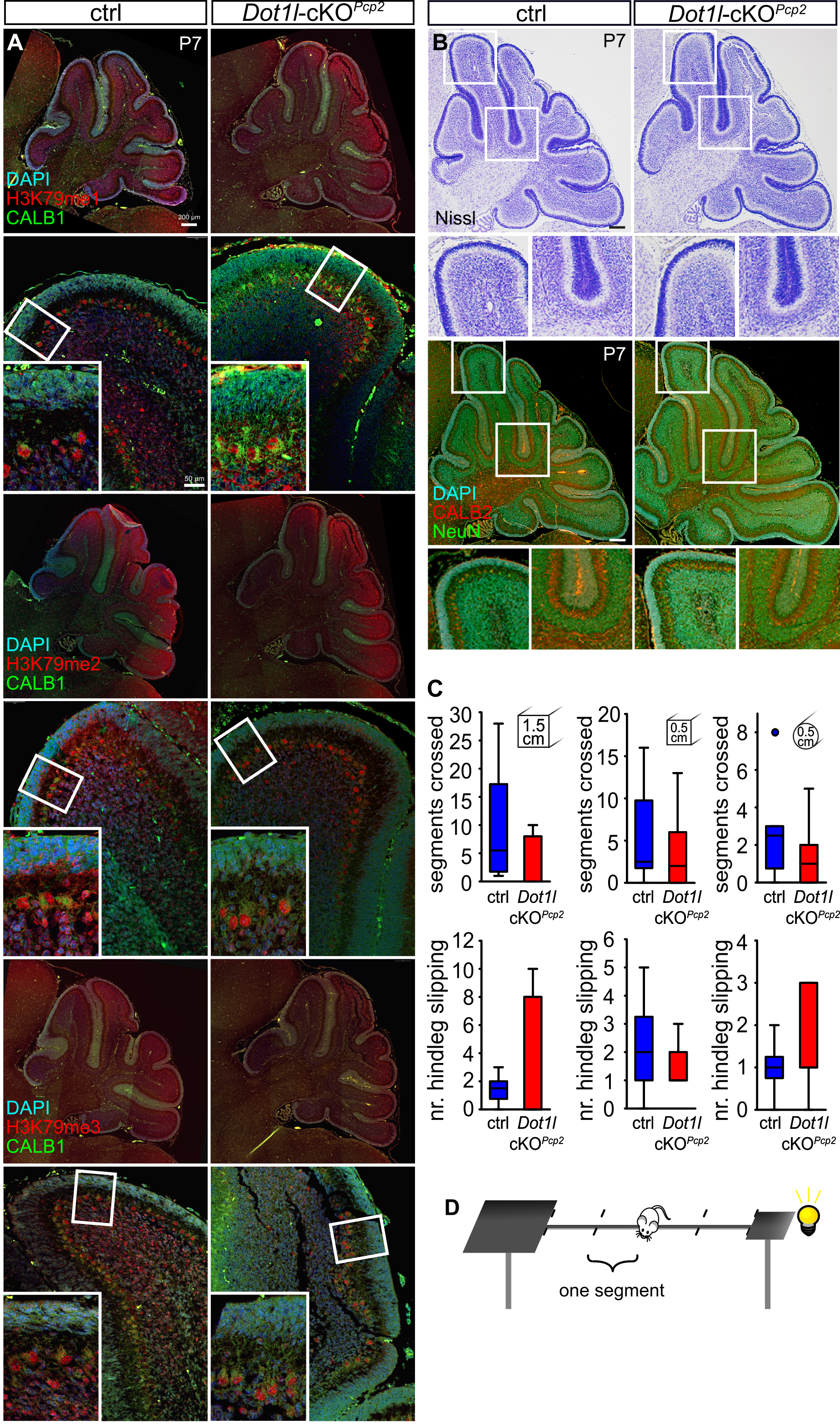

Supplement: Supplementary file 2 — Conditional inactivation of DOT1L does not affect Purkinje cell development in Dot1l-cKOPcp2. (A) Immunostaining of H3K79me1, H3K79me2 and H3K79me3 of P3 control (Dot1lf/f, Pcp2+/+) and Dot1l-cKOPcp2 cerebellum. Scale bars: 200 μm or 50 μm respectively. (B) Nissl stainings and immunostainings of CALB2 and NeuN of P7 old control (Dot1lf/f, Pcp2+/+) and Dot1l-cKOPcp2 mice do not show phenotypic differences. Scale bar: 200 μm. (C) Balance beam motor test with increasing challenge through decreased beam diameters and form: square 1.5 cm (left), 0.5 cm (middle) and round 0.5 cm (right). Control (Dot1lf/f, Pcp2+/+) n = 8 and Dot1l-cKOPcp2n = 5, 9 to 10 W old male mice. Given is the number of segments crossed and of hind leg slipping. Data represented in median whisker-box plots. H0: mutant = ctrl performance, H1: mutant < ctrl performance; one-sided t-test, unpaired, equal variance. H0 cannot be rejected. (D) Schematic drawing of the experimental set-up of the balance beam motor test. (PNG 7973 kb) [file 12035_2018_1377_MOESM2_ESM.png]

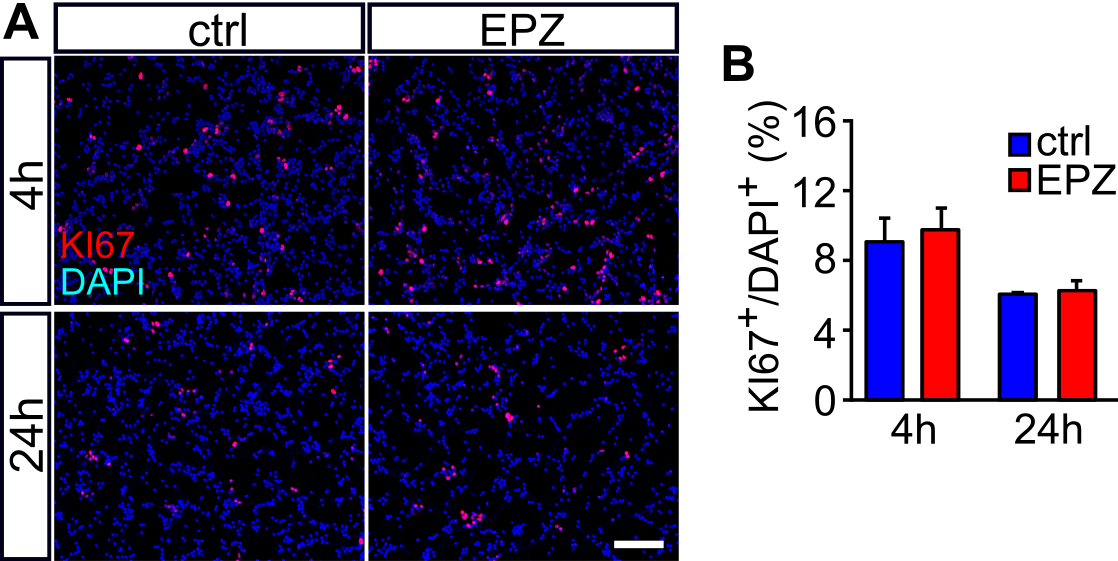

Supplement: Supplementary file 3 — Proliferation of CGNP is not impaired upon DOT1L inhibition with EPZ5676. (A) Immunostaining (ICC) and (B) quantification of KI67 positive CGNP 4 h and 24 h after DOT1L inhibition with EPZ5676 (red bars) and DMSO control (blue bars). Given is the percentage of stained cells per DAPI positive cells. n = 3, mean ± SEM, unpaired two-tailed t-test with equal variance. (PNG 405 kb) [file 12035_2018_1377_MOESM3_ESM.png]

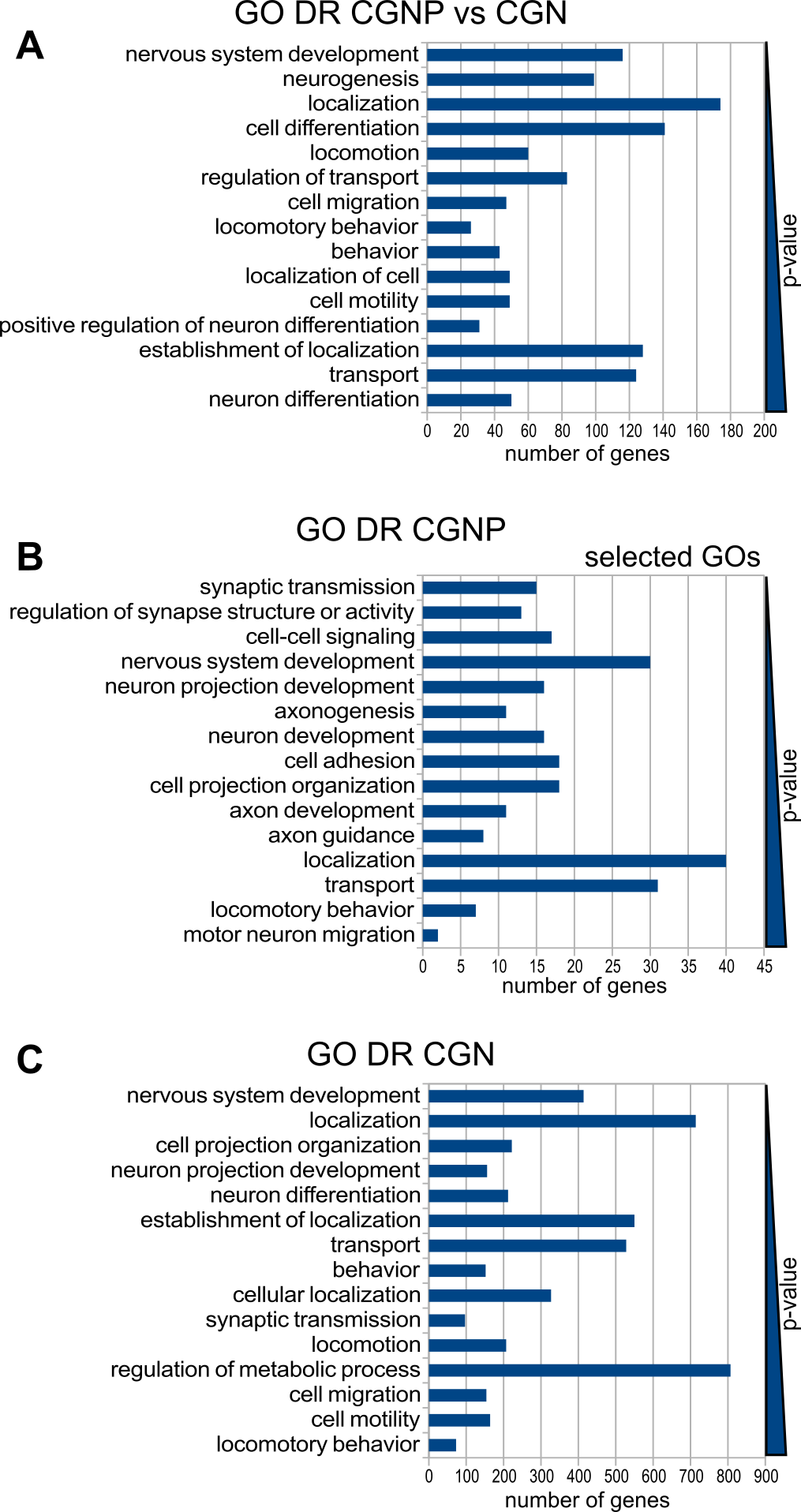

Supplement: Supplementary file 4 — GO term analysis for biological processes of differentially methylated (DR) genes. (A-C): Selected GO terms associated to locomo*, migra*, locali*, motil*, cycle*, metabol*, cholest*, lipid*, transport*, stress*, neuro*, cerebell*, projection, axon, dendri* and related terms. First among the 100 most significant terms were preferentially selected. Arrangement from top to bottom according to increasing p-value. Bars represent the number of genes falling in each GO term category. (PNG 377 kb) [file 12035_2018_1377_MOESM4_ESM.png]

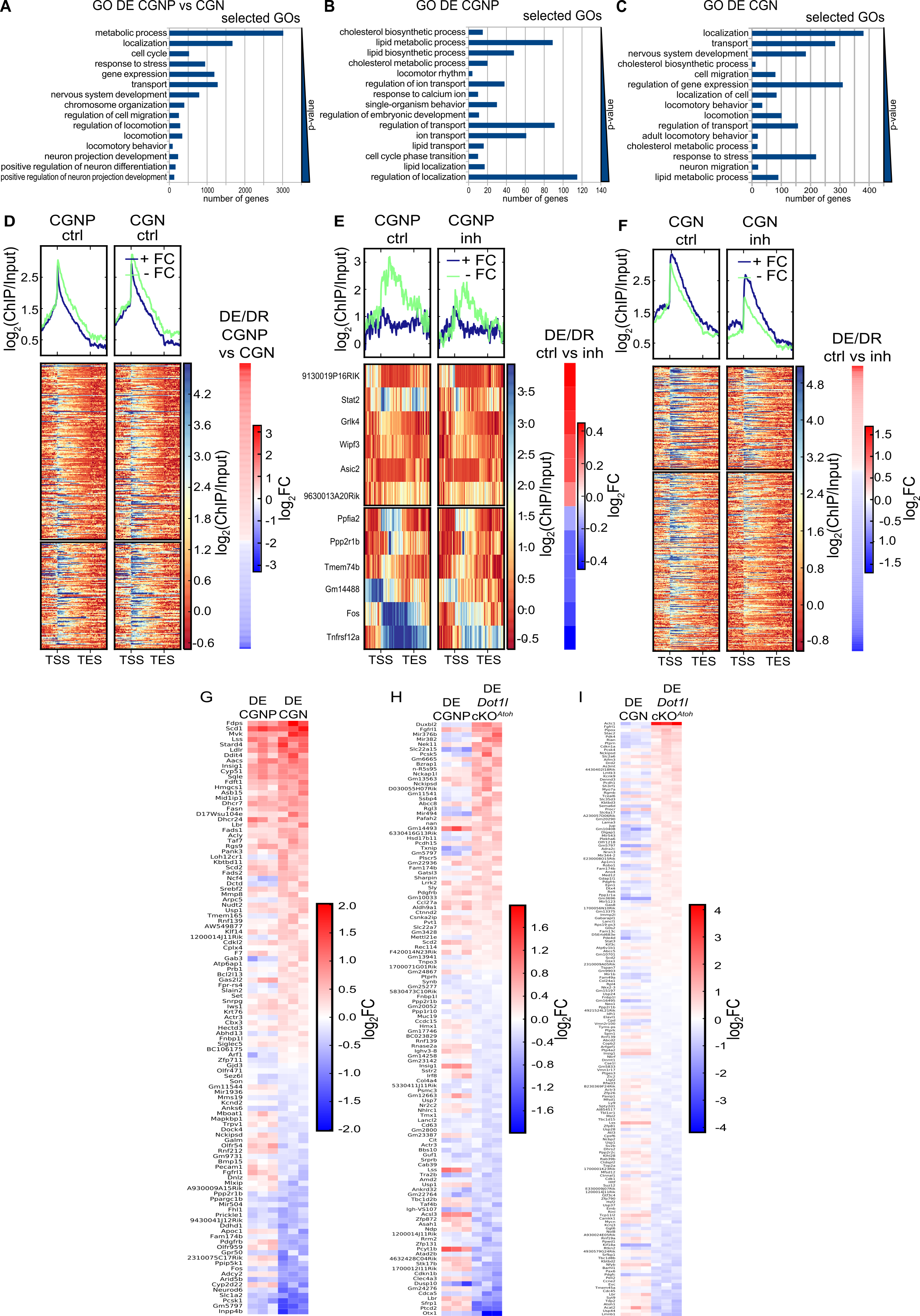

Supplement: Supplementary file 5 — Analysis of differentially expressed genes (DE) in CGNP and CGN upon DOT1L inhibition. (A-C): Selected GO terms associated to locomo*, migra*, locali*, motil*, cycle*, metabol*, cholest*, lipid*, transport*, stress*, neuro*, cerebell*, projection, axon, dendri* and related terms. First among the 100 most significant terms were preferentially selected. Arrangement from top to bottom according to increasing p-value. Bars represent the number of genes falling in each GO term category. (D-F) Mean enrichment (top panels) and heatmaps (lower panels) for DE genes (p-value cut-off: p ≤ 0.05, n = 3) that are as well differentially methylated for H3K79me2 (DE/DR genes). Heatmaps were separated in increased (red) or decreased (blue) differential expression, and sorted after DE level represented as log2FC. (D) For differentiation from CGNP to CGN, (E) CGNP treated with DOT1L inhibitor for 4 h and (F) CGN treated with DOT1L inhibitor for 44 h. (G) Comparison of DE genes in CGNP ctrl vs. inhibitor treatment with DE genes of CGN ctrl vs. inhibitor. (H) Comparison of DE genes in CGNP ctrl vs. inhibitor treatment with DE genes of Dot1l-cKOAtoh1 vs. ctrl cerebellum. (I) Comparison of DE genes in CGN ctrl vs. inhibitor treatment with DE genes of Dot1l-cKOAtoh1 vs. ctrl cerebellum. Cut-off p-value ≤0.05, displayed in log2FC, red: increased expression, blue: decreased expression. (PNG 1965 kb) [file 12035_2018_1377_MOESM5_ESM.png]

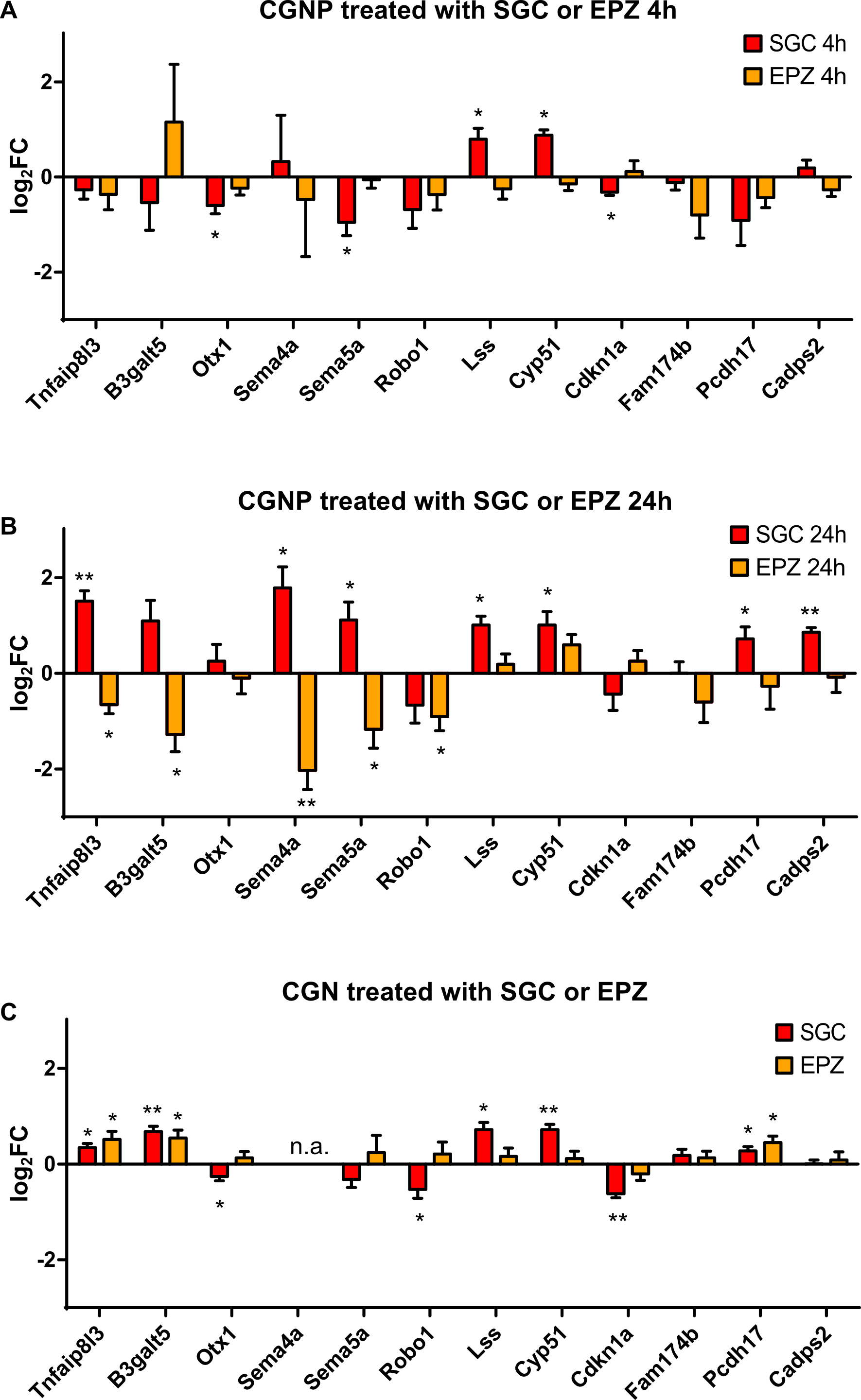

Supplement: Supplementary file 6 — Verification of 12 DOT1L target genes in CGNP and CGN cells treated with inhibitor SGC0946 (SGC) or EPZ5676 (EPZ). (A-C) qRTPCR of putative DOT1L target genes in CGNP treated with DOT1L inhibitors SGC (red bars) and EPZ (orange bars) for 4 h (A) or 24 h (B), and CGN treated with DOT1L inhibitors for 44 h (C). Given is the log2FC ± SEM between SGC0946 or EPZ5676 and controls (n = 3), two-sided t-test, with equal variance. *: p ≤ 0.05, **: p ≤ 0.005, ***: p ≤ 0.0005. (PNG 320 kb) [file 12035_2018_1377_MOESM6_ESM.png]
