# Supplementary material for: Differential Methylation of H3K79 Reveals DOT1L Target Genes and Function in the Cerebellum In Vivo
Source: Mol Neurobiol. 2018 Oct 10;56(6):4273–87. doi: 10.1007/s12035-018-1377-1 (PMC6505521; doi:10.1007/s12035-018-1377-1)
Supplement: Supplementary file 7 — Putative direct DOT1L target genes in the cerebella granule cells. (PDF 25 kb) [file 12035_2018_1377_MOESM7_ESM.pdf]

		Microarray			qRT-PCR			Microarray			Microarray		
		Dot1l -cKO vs wt			*Dot1l* -cKO vs wt			CGNP DMSO vs SGC			CGN DMSO vs SGC		
	**Function**	**log2(fold change)**	**SEM**	**p-value**	**log2(fold change)**	**SEM**	**p-value**	**log2(fold change)**	**SEM**	**p-value**	**log2(fold change)**	**SEM**	**p-value**
Tnfaip8l3	Signaling	-0,92	0,08	0,00	-1,04	0,25	0,00	n/a	n/a	n/a	n/a	n/a	n/a
B3galt5	Signaling	-0,80	0,26	0,04	0,28	0,07	0,00	n/a	n/a	n/a	n/a	n/a	n/a
Otx1	Transcription factor	-1,52	0,45	0,02	-1,16	0,42	0,04	-0,38	0,47	0,04	n/a	n/a	n/a
Sema4a	Cell migration	0,64	0,23	0,02	0,40	0,16	0,05	n/a	n/a	n/a	n/a	n/a	n/a
Sema5a	Cell migration	0,41	0,14	0,03	2,31	0,81	0,02	n/a	n/a	n/a	n/a	n/a	n/a
Robo1	Cell migration	0,30	0,12	0,03	0,21	0,10	0,05	n/a	n/a	n/a	-0,70	0,14	0,01
Lss	Cholesterol and lipid metabolism	-0,28	0,08	0,03	-0,58	0,09	0,00	0,84	0,21	0,01	1,22	0,07	0,01
Cyp51	Cholesterol and lipid metabolism	n/a	n/a	n/a	-0,15	0,06	0,01	0,74	0,06	0,00	0,85	0,06	0,02
Cdkn1a	Cell cycle	0,54	0,12	0,01	0,66	0,06	0,00	n/a	n/a	n/a	-0,44	0,16	0,02
Fam174b	unknown function	0,29	0,11	0,04	0,85	0,39	0,05	-0,12	0,03	0,01	-0,59	0,02	0,00
Pcdh17	Calcium dependent Cell adhesion/Exocytosis	0,52	0,20	0,05	1,56	0,53	0,00	n/a	n/a	n/a	n/a	n/a	n/a
Cadps2	Calcium dependent Cell adhesion/Exocytosis	-0,68	0,11	0,00	-1,52	0,12	0,04	n/a	n/a	n/a	n/a	n/a	n/a
